# The Outcome of Hodgkin Lymphoma With Reference to Prognostic Markers

**DOI:** 10.7759/cureus.28421

**Published:** 2022-08-26

**Authors:** Rehana Ahmed, Faryal Tariq, Javeria Ashfaq, Warkha Thakur, Sidra Zafar, Asma Danish, Munira Borhany

**Affiliations:** 1 Clinical Hematology, National Institute of Blood Diseases and Bone Marrow Transplantation, Karachi, PAK; 2 Internal Medicine, Jinnah Postgraduate Medical Centre, Karachi, PAK; 3 Research and Development, National Institute of Blood Diseases and Bone Marrow Transplantation, Karachi, PAK

**Keywords:** radiotherapy (rt), chemotherapy, erythrocyte sedimentation rate, prognostic biomarkers, hodgkin’s lymphoma

## Abstract

Objectives: This study aimed to determine the impact of prognostic markers on the outcomes of Hodgkin lymphoma.

Methods: It is a cross-sectional, single-center study. A total of 60 patients diagnosed with Hodgkin lymphoma were recruited for the study over five years between 2016 to 2020. The study setting was the National Institute of Blood and Bone Marrow Transplant in Pakistan. The Statistical Package for Social Sciences (SPSS) version 23 (IBM Corp., Armonk, NY, USA) was used for statistical analysis.

Results: In the study population, 63.3% of the patients were male (38/60), and 36.7% were female (22/60). Hodgkin lymphoma was divided into four stages: stage I (18.3%), stage II (18.3%), stage III (46.7%), and stage IV (16.7%). Patients in stage III had a higher value of hemoglobin (Hb) than in other stages of the disease. The erythrocyte sedimentation rate was high in 56.7% of stage III patients than in patients of the other stages. The lactate dehydrogenase (LDH) levels were not under the normal range in 51.6% of patients. Only 20% of patients in stage III had LDH values within the normal range, whereas 26.6% did not.

Conclusion: There was a significant impact of prognostic factors on the survival of patients with Hodgkin lymphoma.

## Introduction

Hodgkin lymphoma (HL) is a lymphoid tumor that arises from B-cells in the embryological center or post-germinal center [1]. Thomas Hodgkin was the first to identify the condition in 1832 [2]. In 2018, HL was responsible for 0.4% of clinically diagnosed malignancies globally [3]. The prevalence of HL fluctuates irrespective of age, ethnicity, and location [4,5]. It is a type of malignant tumor in which the cancerous Hodgkin and Reed-Sternberg (HRS) cells are enveloped by extensive cellular infiltration of immune cells (lymphocytes, macrophages, eosinophils, mast cells, plasma cells, and collagen cells) and also contribute to the tumor's microenvironment [6].

Significant improvement in mortality of the disease has been observed due to a variety of chemotherapeutic medicines and low-dose field radiation [7]. One of the most significant medical oncology success stories of the 20th century is the treatment of HL. Patients' outcomes are influenced by a variety of factors, including their condition and the possibility of short- and long-term therapeutic side effects. Chemotherapy is the cornerstone of classic Hodgkin lymphoma (cHL) treatment. Owing to effective chemotherapeutic treatment, and the improvement of radiotherapy technology, mortality has improved throughout the millennia. The five-year life expectancy of the condition is 80% to 97.3% attributable to initial chemotherapy doxorubicin hydrochloride (Adriamycin), bleomycin sulfate, vinblastine sulfate, and dacarbazine (ABVD), and escalating bleomycin sulfate, etoposide phosphate, doxorubicin hydrochloride (Adriamycin), cyclophosphamide, vincristine sulfate (Oncovin), procarbazine hydrochloride, and prednisone (BEACOPP) treatment [8-11].

Identifying patients who are likely to fail to amend the existing prognosis assessment is critical. For vulnerability assessment and directed therapeutic regimens, the international prognostic score (IPS) and interim positron emission tomography (PET) were previously used [12-14]. Early progression of disease (POD, disease progression within 12 months of induction) has, on the other hand, been identified as a high-risk sign [15]. Prognostic factors of HL can be split into three categories: disease-associated factors, factors connected to the patient as a disease host, and factors that contribute to the treatment. There are interconnections between such domains. The patient's genetic history is an important factor that affects the metabolism of cytotoxic medications, the treatment's effectiveness, and adverse reactions. Prognostic indicators aid in the development of risk-based treatment regimens and the identification of patients who are at risk of failing [16]. Hodgkin lymphoma is classified into preliminary phase and later stage malignancy based on these diagnostic variables. Participants with favorable prognostic characteristics have been observed for combined modality therapy and reported fewer chemotherapy cycles, lower radiotherapy (RT) field dosages, and even chemotherapy solely [17]. Therefore the goal of this study was to determine the impact of prognostic markers on the outcomes of Hodgkin lymphoma.

## Materials and methods

A cross-sectional single-center study was conducted at the National Institute of Blood Disease and Bone Marrow Transplantation. The study comprised 60 hospitalized patients with positive HL disease between 2016 to 2020. Data was collected after approval from the Institutional Review Board (IRB) of the National Institute of Blood Disease and Bone Marrow Transplantation, Pakistan (approval no. NIBD/RD-227/13-2019). All the participants (or their parents/guardians) gave their written informed consent. After evaluating the files of histopathology, information on patients with HL was analyzed, and patients were then contacted for a follow-up discussion. Age, gender, clinical stage, histopathology evaluation, B-symptoms, evidence of treatment, serum lactic dehydrogenase level, erythrocyte sedimentation rate (ESR), relapse, hematological profile (platelet counts, and hemoglobin), and the number of extranodal sites of the malady were used to calculate the biomarker measurement. The Ann Arbour staging system was used to stage the patients i.e., stages I, II, III, and IV. Patients were given standard chemotherapy treatments, relapse, salvage (Dexa-BEAM), transplantation, and radiation. While interviewing the patients, B-symptoms were also noted. All the patients were treated and given two to four, or six to eight cycles of ABVD, accompanied by re-assessment imaging by PET scan and a discussion about radiotherapy in a comprehensive conference if the metabolic or morphological response was insufficient and If the ESR was greater than 30mm/hour and the bulk volume was greater than 200ml. Patients who did not have a formal pathology report in their hospital documents in digital form were not included in the study. The Statistical Package for Social Sciences (SPSS) version 23 (IBM Corp., Armonk, NY, USA) was used for statistical analysis. Proportions were utilized for explanatory data; mean and standard deviation were used for variable data. The categorical data were analyzed using the chi-square or Fisher exact test (where needed), and an independent t-test was employed to examine the mean difference between continuous variables.

## Results

**Figure 1:**
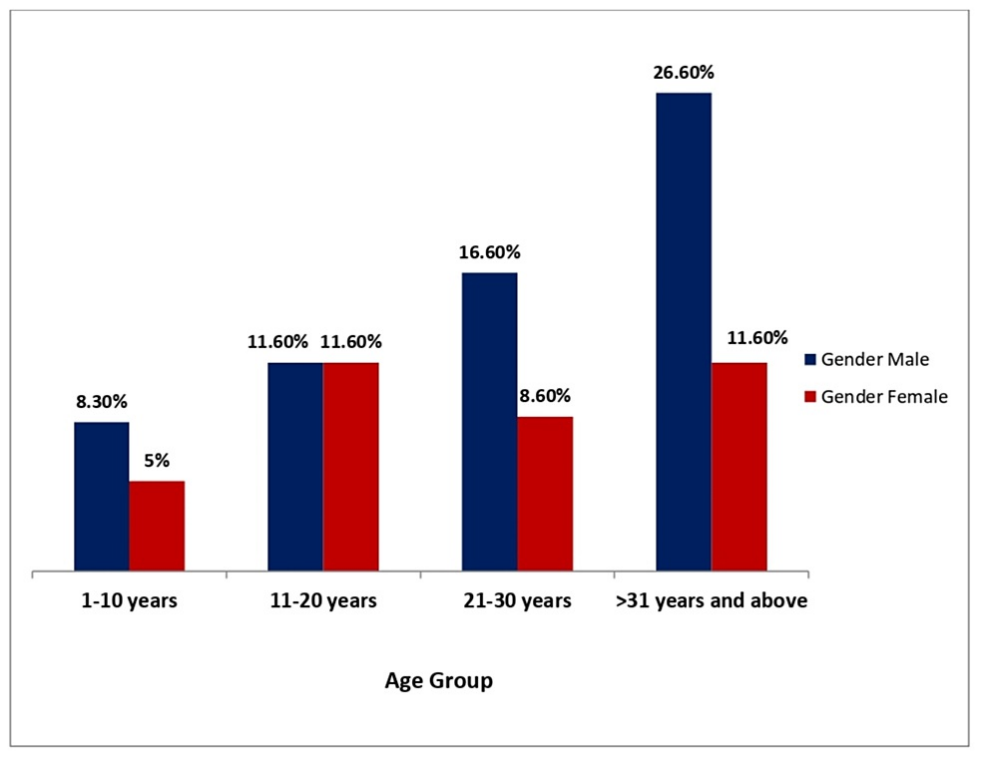
Graphical explanation of age groups and gender distribution in the study

Gender distribution (male and female) according to age groups is presented in Figure 1. Age groups include one to 10 years as group I, 11 to 20 years as group II, 21 to 30 years as group III, and 31 or more years as group IV. In groups I, III, and IV, the distribution of males were higher than the females whereas in group II the distribution of males and females was equal.

The frequency of patient characteristics using descriptive analysis is presented in Table 1. According to gender distribution, most of the patients were male i.e., 38 (63.3%) while 22 were female (36.7%). The B-symptoms were positive in 70% of patients, however, 46.7% of the patients were diagnosed with stage III HL. Further details are presented in Table 1.

**Table 1:** Frequency of patient characteristics

Variables	Characteristics	Frequency n (%)
Gender distribution	Male	38 (63.3)
	Female	22 (36.7)
B-symptoms	Negative	18 (30)
	Positive	42 (70)
Classifications	Nodular sclerosis	28 (43.3)
	Mixed cellularity	26 (43.3)
	Lymphocyte rich	6 (10)
	Lymphocyte depletion	2 (3.3)
Stages	I	11 (18.3)
	II	11 (18.3)
	III	28 (46.7)
	IV	10 (16.7)
Bulk volume	No	31 (51.6)
	Yes	29 (48.3)

The association of treatment given to the patients with HL and disease staging is presented in Table 2 using bivariant analysis. All of the therapeutic modalities including relapse, salvage, chemotherapy, and radiation therapy showed significant p-values. Most of the patients in advanced stages (stage III and stage IV) received increased treatment.

**Table 2:** Association of therapeutic modalities with disease staging *significant ≤0.05 P-value ABVD: Doxorubicin hydrochloride (Adriamycin), bleomycin sulfate, vinblastine sulfate, and dacarbazine; BEACOPP: Bleomycin sulfate, etoposide phosphate, doxorubicin hydrochloride (Adriamycin), cyclophosphamide, vincristine sulfate (Oncovin), procarbazine hydrochloride, and prednisone; OEPA: Vincristine sulfate (Oncovin), etoposide, prednisone, and doxorubicin hydrochloride (Adriamycin)

Variables	Characteristics	Frequency n (%)	Stage I, 11 (18.3%)	Stage II, 11 (18.3%)	Stage III, 28 (46.7%)	Stage IV, 10 (16.7%)	P-Value
Chemotherapy	2-4 Cycles ABVD	33 (55)	8 (24.2)	7 (21.2)	14 (42.5)	4 (12.1)	0.05*
	6-8 Cycles ABVD	24 (40)	3 (12.5)	4 (16.7)	12 (50)	5 (20.8)	
	2-6 Cycles OEPA	1 (1.7)	0 (0)	0 (0)	1 (100)	0 (0)	
	2-6 Cycles BEACOPP	2(3.3)	0 (0)	0 (0)	1 (50)	1 (50)	
Relapse	No relapse	26 (43.3)	7 (26.9)	3 (11.5)	11 (42.3)	5 (19.2)	0.04*
	Relapse	34 (56.7)	4 (11.8)	8 (23.5)	17 (50.0)	5 (14.7)	
Salvage	No	12(35.3)	3 (25)	1 (8.4)	5 (41)	4 (33.4)	0.04*
	Yes	22 (64.7)	6 (27.3)	3 (13.7)	8 (36.4)	5 (22.6)	
Transplantation	Yes	16 (72.8)	4 (25)	5 (31.2)	6 (37.5)	1 (6.3)	0.60
	No	6 (27.2)	1 (16.6)	0 (0)	3 (50)	2 (33.4)	
Radiation therapy	Yes	7 (11.6)	1 (14.2)	0 (0)	4 (57.1)	2 (28.5)	0.02*
	No	53 (88.4)	10 (18.9)	11 (20.7)	24 (45.3)	8 (15.1)	

The association of the prognostic factors of HL patients was determined using bivariant analysis and is presented in Table 3. Low-level hemoglobin (Hb) (<11) was observed in 28.3% of patients, 66.7% had normal Hb (11.6-16.6), and 5% of patients showed high levels of Hb (above 16.6). However, patients in stage III had a higher value of Hb as compared to other stages. Erythrocyte sedimentation rates were high in 56.7% of stage III patients than in other stages. The lactate dehydrogenase (LDH) level was not under the normal range in 51.6% of patients while 48.3% of patients had a normal range of LDH. In stage III, LDH was under the normal range with only 20% of patients while 26.6% of patients showed a deranged value of LDH. Similarly, a normal range of platelet count was only present in 73.3% of patients while 11.6% of patients had a low level of platelets and 15% of patients showed a high level of platelet count.

**Table 3:** Association of prognostic factors of HL patients with disease staging *significant ≤0.05 P-value LDH: Lactate dehydrogenase, Hb: Hemoglobin, CD: Cluster of differentiation

Variables		Frequency n (%)	Stage I, 11 (18.3%)	Stage II, 11 (18.3%)	Stage III, 28 (46.7%)	Stage IV, 10 (16.7%)	P-value
Erythrocyte sedimentation rate	Less than 30	26 (43.3)	3 (11.5)	4 (15.4)	14 (53.8)	5 (19.2)	0.18
	Above 30	34 (56.7)	8 (23.5)	7 (20.6)	14 (41.2)	5 (14.7)	
LDH level	Normal range (140 to 280 units/L)	29 (48.3)	6 (20.7)	7 (24.1)	12 (41.4)	4 (13.8)	0.30
	Abnormal range (greater than 280 units/L)	31 (51.7)	5 (16.1)	4 (12.9)	16 (51.6)	6 (19.4)	
Hemoglobin level (g/dL)	Low-level HB (<11)	17 (28.3)	3 (17.6)	1 (5.9)	10 (58.8)	3 (17.6)	0.38
	Normal HB (11.6- 16.6)	40 (66.7)	7 (17.5)	10 (25.0)	16 (40)	7 (17.5)	
	High-level HB (above 16.6)	3 (5)	1 (33.3)	0 (0)	2 (66.7)	0 (0)	
CD30	Positive	57 (95)	9 (15.8)	11 (19.3)	27 (47.4)	10 (17.5)	0.00*
	Negative	3 (5)	2 (66.7)	0 (0.0)	1 (33.3)	0 (0.0)	
CD15	Positive	56 (93.3)	9 (16.1)	11 (19.6)	26 (46.4)	10 (17.9)	0.00*
	Negative	4 (6.7)	2 (50)	0 (0)	2 (50)	0 (0)	
Platelet count	Low level (below 150 per microliter)	7 (11.6)	2 (28.6)	1 (14.3)	2 (28.6)	2 (28.6)	0.441
	Normal (150-450per microliter)	44 (73.3)	9 (20.5)	8 (18.2)	20 (45.5)	7 (15.9)	
	High level (above 450 per microliter)	9 (15)	0 (0)	2 (22.2)	6 (66.7)	1 (11.7)	

## Discussion

Clinical and histological characteristics of patients with HL differ across industrialized and developing nations. This study examined the predictive indicators for HL in patients from Pakistan. An epidemiological study by Mathas et al. [18] revealed that HL had an age distribution. According to our analysis, the overall incidence of HL was observed between the ages of 11 and 30. Overall, there were 63.30% more male patients in the research sample than female patients (36.70%). However, a study conducted by Ghafoor et al, [19] in Pakistan reported a 79.2% incidence of HL in males as compared to females (20.80%). The findings were consistent with those of our investigation. This distribution resembles that of industrialized nations. Hodgkin lymphoma is further categorized by clinical criteria present at diagnoses, such as stage of disease, bulk volume, and presence of systemic symptoms, as explained by McMahon [20] in his study, whereas Kelly [21] reported the prognostic factors and the outcomes of primary care treatment in 5141 cases of advanced-stage HL from 23 centers.

According to our research, several factors are important for predicting patient outcomes. Acute phase reactant erythrocyte sedimentation rate (ESR), which has prognostic importance in individuals with Hodgkin lymphoma, can have a range of baseline levels before being considered significant [22]. An ESR of more than 30 mm/hr in Euronet-pediatric Hodgkin lymphoma (PHL) is important. Around 56.7% of the participants in our study had an ESR greater than 30 mm/hr. In a Pakistani study, Sindhu et al. [23] showed that 80% of patients in the advanced stage of the disease had an ESR greater than 30 mm/hr and a significant p-value of 0.003. This study and ours were comparable because we both reported significant p-values of 0.00. The B-symptoms were present in 10% to 25% of patients with early-stage cancer and up to 70% of patients with advanced-stage disease, according to a study published by Cuccaro et al. [24]. In our study, the B-symptoms were present in 45.2% of cases in stage III (advanced stage), and 23.8% of cases in stage I (early disease stage). According to Cellini et al., it was found in 22% of patients with early disease, and 49% of patients with late disease [25]. The results of our investigation showed that bulk volume was not a major risk factor. The study by Sindhu [23] supported our data and showed that bulk volume was not recognized as a risk factor for HL. Many hospitals utilize low-dose radiation on patients who have refractory or partial responses to chemotherapy to lower the risk of subsequent cancers [26]. Patients in our research received radiation at a rate of 11.6%, thus results from our investigation are very much similar to other studies. A previous study from Pakistan reported that radiotherapy was used for 15% of the patients [27].

With typical second-line regimens, patients who either are resistant to or relapse after initial combination chemotherapy have around a 20% chance of being cured [28]. One hundred seven patients who had relapsed were retrospectively examined by Longo et al. [28]. In their study, 94 patients received mechlorethamine hydrochloride, vincristine sulfate (Oncovin), procarbazine hydrochloride, and prednisone (MOPP) as their main treatment plan, whereas 13 patients received MOPP/ABVD. Less than 10% of these relapses happened five years after chemotherapy, but half of them happened within a year of finishing treatment. In the Pakistani study by Sindhu [23], 95.2% of patients did not have a recurrence. In our study, 56.7% of the participants had relapsed. Therefore, our findings were different compared to earlier studies [25,26-28].

A study by Belgaumi et al. reported less than 80% of patients had HL, 94% were diagnosed with early stage, and 96% were late stage patients [29]. For patients of early- and advanced-stage HL, a variety of prognostic variables were identified: bulky disease, ESR, LDH, hemoglobin, and the presence of B-symptoms, with age being the most important one [30]. These prognostic indicators are taken into account when developing treatments for HL cases in the early and advanced stages. Stage I and II cases made up 18.30% of all HL cases in our study. Additionally, patients with poor prognostic markers such as B-symptoms and bulky disease had high blood LDH and ESR levels. Additionally, those same patients with poor prognostic indicators had lower rates of other prognostic factors including platelets and hemoglobin. In the overall analysis of our study, the survival of the early- and advanced-stage HL cases was affected by the determinants including the stage of the disease, the presence of B-symptoms, radiation, ESR, serum LDH level, and platelets. When characteristics identified as having prognostic relevance in the univariate study were reevaluated using multivariate analysis, it was discovered that a high LDH level and the presence of B-symptoms still had significant prognostic implications. These outcomes matched those from earlier studies.

The limitations of our study include the small sample size and the limited number of cases in our population since it is a single-center study. Therefore, multi-center longitudinal studies should be conducted in the future for a better understanding of the disease outcome.

## Conclusions

Prognostic markers predict outcomes with the ultimate goal of achieving a customized therapeutic approach. Over the past 10 years, several new prognostic variables for HL have been discovered in Pakistan. It is concluded that the survival of the patients in early- and advanced-stage HL was affected by the determinants, including the stage of the disease, the presence of B-symptoms, ESR, serum LDH level, and platelets. It was discovered that a high LDH level and the presence of B-symptoms still had significant prognostic implications. We consider our study's findings to be noteworthy and significant. Overall, influential research in this aspect and other scale studies must be conducted in the region.
